# Development of a Critical Nitrogen Dilution Curve Based on Leaf Area Duration in Wheat

**DOI:** 10.3389/fpls.2017.01517

**Published:** 2017-09-04

**Authors:** Xiaolong Wang, Tianyang Ye, Syed Tahir Ata-Ul-Karim, Yan Zhu, Leilei Liu, Weixing Cao, Liang Tang

**Affiliations:** ^1^National Engineering and Technology Center for Information Agriculture, Key Laboratory for Crop System Analysis and Decision Making, Ministry of Agriculture, Jiangsu Key Laboratory for Information Agriculture, Jiangsu Collaborative Innovation Center for Modern Crop Production, Nanjing Agricultural University Nanjing, China; ^2^Henan Institute of Science and Technology Xinxiang, China; ^3^Key Laboratory of Soil Chemistry and Environmental Protection, Institute of Soil Science, Chinese Academy of Sciences Nanjing, China

**Keywords:** wheat, leaf area duration, critical nitrogen dilution curve, nitrogen nutrition index, yield

## Abstract

Precise quantification of plant nitrogen (N) nutrition status is essential for crop N management. The concept of critical N concentration (Nc) has been widely used for assessment of plant N status. This study aimed to develop a new winter wheat Nc dilution curve based on leaf area duration (LAD). Four field experiments were performed on different cultivars with different N fertilization modes in the Yangtze River basin and Yellow River basin in China. Results showed that the increase in LAD with increasing cumulative thermal time took the shape of an “S” type curve; whereas shoot N concentration decreased with increasing LAD, according to a power function. Both LAD and shoot N concentration increased with increasing N application. The new LAD based Nc dilution curve was determined and described as Nc = 1.6774 LAD^−0.37^ when LAD > 0.13. However, when LAD ≤ 0.13, Nc was constant and can be calculated by the equation when LAD = 0.13. The validation of Nc dilution curve with dataset acquired from independent experiments confirmed that N nutrition index (NNI) predictions based on the newly established Nc dilution curve could precisely diagnose N deficiency at different plant growth stages. The integrated N nutrition index (NNI_inte_), which was obtained by the weighted mean of NNI, was used to estimate shoot N concentration, shoot dry matter, LAD, and yield using regression functions. The linear relationships between NNI_inte_ and these growth variables were well correlated. These results provided enough evidence that the new LAD–based Nc dilution curve could effectively and precisely diagnoses N deficiency in winter wheat crops.

## Introduction

Nitrogen (N) is a major nutritional element required for crop growth and productivity (Eickhout et al., 2006). In-season N diagnosis and management in crop production is an important agronomic practice for ensuring food security and environmental sustainability (Ata-Ul-Karim et al., 2016a,b). N is an integral component of chlorophyll and protein related with plant vigor, yield and quality and appropriate N fertilizer application is not only imperative for improving crop production but also for increasing the processing quality of grain in cereals (Wang et al., 2002; Zhu et al., 2003). Previous studies have shown a strong relationship between N status and wheat yield and quality. Excessive N fertilizer application has negative impacts, such as decreased N use efficiency and deterioration of the ecological environment (Jeuffroy and Meynard, 1997; Zhao et al., 2016a). Therefore, accurate estimation of crop N status and precision N management has become pressing study topics for crop growers and scientists.

Reliable estimation of crop N requirement and diagnosing nutritional status at critical crop growth stages is imperative for optimizing the quantitative and qualitative aspects of crop production (Ata-Ul-Karim et al., 2016b). Non-destructive diagnostic tools such as chlorophyll meter, digital camera, active and passive sensors (Mistele and Schmidhalter, 2008; Lee and Lee, 2013; Yuan et al., 2016) have been widely used to assess the crop N status. Besides non-destructive diagnostic tools, the concept of critical N (Nc), which is crop specific, simple and biologically sound diagnostic tool, has also widely been used as a classical destructive diagnostic tool for diagnosing crop N status. The Nc dilution curves based on shoot biomass have been established in various agronomic crops, including corn (Plénet and Lemaire, 1999; Ziadi et al., 2008; Yue et al., 2014), rice (Sheehy et al., 1998; Ata-Ul-Karim et al., 2013; Huang et al., 2015; He et al., 2017), wheat (Justes et al., 1994; Ziadi et al., 2010; Yue et al., 2012; Zhao et al., 2012). Attempts have also been made to establish the Nc dilution curves on organ specific dry matter (leaf dry matter and panicle dry matter) in wheat (Yao et al., 2014a; Zhao et al., 2016b), on leaf dry matter and stem dry matter in rice ecotypes (Ata-Ul-Karim et al., 2014a; Yao et al., 2014b), and on leaf dry matter in maize (Zhao et al., 2017). The existing dilution curves based on whole plant and organ specific dry matter were developed for crops grown in a specific region, and the model parameters vary with climate. Additionally, dry matter is generally measured by destructive sampling. Leaf area index (LAI) and canopy coverage is a fundamental variable in agronomic and environmental research have been widely used as reference plant index for monitoring crop growth, predicting grain yield, and optimization of crop management practices (Ata-Ul-Karim et al., 2014b; Wang et al., 2016). With the advances in sensor technologies, it is convenient to measure LAI non-destructively. LAI measurements are more easily performed than quantification of shoot dry matter, providing an alternative method for evaluation of plant N status diagnosis. Previous studies have developed Nc dilution curves based on LAI, providing an alternative method for plant Nc assessment and N status diagnosis (Confalonieri et al., 2011; Ata-Ul-Karim et al., 2014b; Zhao B. et al., 2014). Crop dry matter accumulation occurs through photosynthetic production, which is depends heavily on the canopy leaves characteristics. Leaf area, its duration, and photosynthetic rate are the key factors driving matter accumulation and yield (Gastal and Bélanger, 1993; Hammer and Wright, 1994; Zhao et al., 2005). Previous studies have shown that N allocation to leaves for growth and photosynthesis to be prioritized within wheat plants (Jeuffroy et al., 2002). N nutritional status, leaf area, and leaf area duration affect both short-term growth status and future productivity. LAI is an instantaneous estimate of the photosynthetic area. However, LAI does not represent photosynthesis as a continuous process occurring within the photosynthetic area and does not precisely explain the formation of dry matter. Leaf area duration (LAD) is the integral of LAI and growth period, and comprehensively incorporates the size and duration of leaf area (Peltonen-Sainio et al., 2008). Therefore, compared with LAI, LAD better represents the process of dry matter accumulation and incorporates plant N dilution mechanisms.

The N diagnostic tools such as N nutrition index (NNI), accumulated N deficit (AND) and N requirement (NR) could be derived from the Nc dilution curve for quantifying the N status, developmental processes and radiation-use efficiency of the plant in response to N supply (Mills et al., 2009; Ata-Ul-Karim et al., 2017a,b) as well as for making decisions on N application (Lemaire et al., 2008). NNI is one of the most widely used Nc curve based N diagnostic tool for in-season plant N status diagnosis (Zhao et al., 2017). The relationships between NNI and relative yield have been previously implicated in assessing yield potential in wheat, corn (Ziadi et al., 2008, 2010), and rice (Ata-Ul-Karim et al., 2016a). Moreover, these relationships have also been used to estimate crop NR for a corrective N fertilization during crop growth period and for assessment of grain amylose and protein content in rice (Ata-Ul-Karim et al., 2017a,c). This concept can successfully differentiate the sub-optimal and supra-optimal N growth conditions in crop production, and the diagnostic tools derived from these curves could be integrated with crop simulation models to assist crop N management (Lemaire et al., 2008; Ata-Ul-Karim et al., 2017b).

The objectives of this study were to develop a Nc dilution curve based on LAD for winter wheat which combines the merits of shoot dry matter- and LAI-based Nc curves and to test the curve through evaluation of the effects of N deficiency on growth variables and yield. The projected results would provide an alternative diagnostic tool for assessing N deficiency limitations to winter wheat growth.

## Materials and methods

### Experiments design

Four experiments at two sites using different cultivars, N levels, split applications, and top-dressing stages were conducted, as summarized in Table 1. Experiment 4 is the historical data obtained from the study of Zhao B. et al. (2014). Urea used as the N fertilizer was applied before sowing (as base fertilizer) and from the jointing to booting stages (as top-dressing fertilizer), respectively. The experimental fields were plowed and subsequently harrowed before sowing. Pre-emergence herbicides were used to control weeds at early growth stages. Plots were also regularly hand-weeded until canopy was closed to prevent weed damage. Insecticides were used to prevent insect damage when necessary. No irrigation was in Experiments 1, 2 and 4. Irrigations (900 m^3^ ha^−1^ per time) at regrowth and jointing were performed in Experiment 3. All other agronomic management practices were used according to local management practices recommendations to ensure maximum potential productivity, i.e., no factor other than N was limiting.

**Table 1:** Basic information of the four experiments.

**Experiment**	**Season**	**Soil characteristics**	**Cultivar**	**N level (Kg ha^−1^)**	**N Topdressing stage and ratio**	**Sampling stages**
Exp. 1 (Yizheng, 32°16′ N, 119°10′ E)	2010-2011	Type: clay soil Organic matter: 14.26 g kg^−1^ Total N: 1.28 g kg^−1^ Available P: 45.46 mg kg^−1^ Available K: 87.23 mg kg^−1^	Yangmai16 (YM16)	0 (N0) 225 (N1) 300 N2	Top 3rd leaf emergence;70%(R1) Top 3rd leaf emergence;60%(R2) Top 3rd leaf emergence;50%(R3) Top 3rd leaf emergence;40%(R4) Top 3rd leaf emergence;30%(R5)	Spring re-growth Jointing Booting Heading Filling
Exp. 2 (Yizheng, 32°16′ N, 119°10′ E)	2010-2011	Type: clay soil Organic matter: 13.54 g kg^−1^ Total N: 1.2 g kg–1 g kg^−1^ Available P: 43.01 mg kg^−1^ Available K: 83.25 mg kg^−1^	Yangmai16 (YM16)	0(N0) 225(N1) 300(N2);	Top 3rd leaf emergence(T1); 60%(R1) Top 3rd leaf emergence(T1); 50%(R2) Top 3rd leaf emergence(T1); 40%(R3) Top 2rd leaf emergence(T2); 60%(R1) Top 2rd leaf emergence(T2); 50%(R2) Top 2rd leaf emergence(T2); 40%(R3) Flag leaf emergence(T3); 60%(R1) Flag leaf emergence(T3); 50%(R2) Flag leaf emergence(T3); 40%(R3)	Spring re-growth Jointing Booting Heading Filling
Exp. 3 (Xinxiang, 35°11′ N, 113°48′ E)	2011-2012	Type: loam soil Organic matter: 15.7 g kg^−1^ Total N: 1.45 g kg–1 g kg^−1^ Available P: 67.54 mg kg^−1^ Available K: 87.43 mg kg^−1^	Aikang58 (AK58)	0(N0) 75(N1) 150 N2 225(N3) 300 (N4)	Top 3rd leaf emergence;65%(R1) Top 3rd leaf emergence;50%(R2) Top 3rd leaf emergence;35%(R3)	Spring re-growth Jointing Booting Heading Filling
Exp. 4 (Yizheng, 32°16′ N, 119°10′ E)	2010-2011	Type: clay soil Organic matter: 13.5 g kg^−1^ Total N: 1.1 g kg–1 g kg^−1^ Available P: 43 mg kg^−1^ Available K: 82 mg kg^−1^	Ningmai13 (NM13)	0(N0) 75(N1) 150 N2 225(N3) 300 (N4) 375 (N5)	Top 3rd leaf emergence;50%	Spring re-growth Jointing Booting Heading

Experiment 1 followed a split plot design, with N level as the main plot factor and N splits as the subplot factor. The study was conducted in Yizheng, China (32°16′ N, 119°10′ E) during the 2010–2011 wheat growing season. Cultivar Yangmai 16 (YM16, grain protein 14.2%) was planted on October 18th by row seeding, with a row spacing of 25 cm. Planting density was 2.40 × 10^6^ plants ha^−1^. P (135 kg ha^−1^ P_2_O_5_) and K (190 kg ha^−1^ K_2_O) fertilizers were incorporated into the soil as monocalcium phosphate (Ca(H_2_PO_4_)_2_) and potassium chloride (KCl) before planting.

Experiment 2 applied a split-split plot design with N level as main plot factor, N split applications as the subplot factor and N application stage as the sub-sub plot factor. The experiment was conducted in Yizheng, China (32°16′ N, 119°10′ E) during the 2010–2011 wheat season. Cultivar Yangmai 16 (YM16, grain protein 14.2%) was planted on November 7th by row seeding, with a row spacing of 25 cm. The area of each plot was 24 m^2^. Planting density was 2.40 × 10^6^ plants ha^−1^. Phosphatic (120 kg ha^−1^ P_2_O_5_) and potassium (150 kg ha^−1^ K_2_O) fertilizers were incorporated into the soil as monocalcium phosphate (Ca(H_2_PO_4_)_2_) and potassium chloride (KCl) before planting.

Experiment 3 was a split plot design with N level as the main plot factor, and N split applications as the subplot factor. The experiment was conducted in Xinxiang, China (35°11′ N, 113°48′ E) during the 2011–2012 wheat growing season. Cultivar Aikang 58 (AK58, grain protein 14.48%) was planted on November 7th by row seeding, with a row spacing of 25 cm. The area of each plot was 24 m^2^. Planting density was 3.0 × 10^6^ plants ha^−1^. Phosphatic (150 kg ha^−1^ P_2_O_5_) and potassium (150 kg ha_−1_ K_2_O) fertilizers were incorporated into the soil as monocalcium phosphate (Ca(H_2_PO_4_)_2_) and potassium chloride (KCl) before planting.

Experiment 4 was conducted involving N levels in Yizheng, China (32°16′ N, 119°10′ E) during the wheat growing season of 2010–2011. Cultivar Ningmai 13 (NM13, Grain protein 10.9%) was planted on 11th Nov. by row seeding, and row spacing was 25 cm. Random plot design was adopted with three replications. Six N levels including 0 (N0), 75(N1), 150(N2), 225(N3), 300(N4), and 375(N5) kg N ha^−1^, were tested, with 50% of the N applied at pre planting and 50% at jointing. The area of each plot was 30 m^2^. Planting density was 2.40 × 10^6^ plants ha^−1^. Phosphatic (96 kg ha^−1^ P_2_O_5_) and potassium (120 kg ha^−1^ K_2_O) fertilizers were incorporated into the soil before planting as monocalcium phosphate (Ca(H_2_PO_4_)^2^) and potassium chloride (KCl).

### Plant sampling, N determination, and daily temperature

Twenty representative plants were sampled from each plot at regrowth, jointing, booting, heading, anthesis, grain filling, and maturity for determination of LAI, shoot dry matter, and N concentration. For shoot dry matter measurements, samples were dried in a forced-draft oven at 80°C until constant weight was achieved. Shoot N was determined following the micro-Kjeldahl method (Bremner and Mulvancy, 1982). LAI was measured using an LI-3000 (Li-COR, Lincoln, US). Daily temperature measurements were acquired from a weather station near the experimental plots.

### Data processing

#### Statistical analysis

The data for determination of critical N points were analyzed according to the methodology proposed by Justes et al. (1994). For each sampling date, experiment and wheat cultivar, the amounts of LAD and plant N concentration were subjected to analysis of variance (ANOVA) using GLM procedures in IBM SPSS Version19.0 (IBM Corporation, Armonk, New York). Significant effects of treatment on mean LAD were tested for using the least significant difference (LSD0.05) test, and the results were used to classify treatments as N-limiting or non-N-limiting. An allometric function was used to determine the relationship between the observed decreases in N concentration with increasing LAD using Microsoft Excel 2010 (Microsoft Corporation, Redmond, WA, USA).

#### Relative growing degree days

In order to account for variation in thermal time among different years and locations, relative growing degree days (RGDD, d°C) was expressed as the ratio of growing degree days (GDD, above 0°, d°C) at a sampling time point to the total GDD across the entire growth period (TGDD, d°C).

(1) RGDD = GDD/TGDD

To perform real-time measurements, the average TGDD from previous years or a real-time TGDD estimated from real-time weather and historic weather data could be substituted for in-season TGDD (Bannayan and Hoogenboom, 2008).

#### Leaf area duration

Leaf area duration (LAD) was calculated using the LAI at each sampling date and the corresponding RGDD according to the equation:

(2) $$\text{LAD}=\int_{0}^{\text{t}}LAI(dt)$$

The LAD between two sampling time points was approximated according to the equation:

(3) $$\text{LAD}=\left({\text{LAI}}_{1}\;+\;{\text{LAI}}_{2}\right)/2\times \left(t_{2}-t_{1}\right)$$

where t_1_ and t_2_ are the sampling times across which RGDD was quantified. LAI_1_ and LAI_2_ are the LAI at the sampling time t_1_ and t_2_, respectively.

#### Establishment and validation of critical nitrogen dilution curve

Data from experiment 1 and 3 were used to develop the Nc dilution curve following the computation method of Justes et al. (1994). If at the same measurement date, statistical analysis distinguished at least one set of N limiting growth and non-N-limiting growth data points, these data were used to define the Nc dilution curve. For each measurement date, the variation in total N concentration and LAD was assimilated into a bilinear relation composed of (a) an oblique line showing the increase in joint LAD and N concentration and (b) a vertical line corresponding to the increase in N concentration and no variation in LAD. The Nc dilution curve was validated for N-limiting and non-N-limiting situations within the range for which it was established using the data collected from the independent experiments (Experiment 2 and 4).

#### Determination of NNI and integrated NNI

The NNI at each sampling date was calculated according to the equation (Justes et al., 1994):

(4) NNI=Nac/Nc

where Nac and Nc, respectively, are the actual N concentration and critical N concentration of the shoot expressed in % dry matter. If NNI = 1, N nutritional status was considered to be optimum, while NNI > 1 and NNI < 1 indicated excess and deficient N nutrition, respectively.

An integrated NNI can be obtained by the weighted mean of NNI (Lemaire et al., 2008) as illustrated as follows.

(5) $${\text{NNI}}_{\text{inte}}=1/T\sum {\text{NNI}}_{\text{i}}\times t_{\text{i}}$$

where NNI_inte_ is the integrated NNI, T represents the duration sampling occurred over (days or GDD), NNI_i_ is the instantaneous NNI value for sampling time point *i*, and t_i_ is the time interval between sampling time point *i* and sampling time point *i*-1.

## Results

### Leaf area duration and shoot N concentration in wheat under different N treatments

Table 2 showed the increase in LAD and the decreases in shoot N concentration (Nac) with the development stages in winter wheat cultivars. Although the four experiments were conducted under different cultivars, treatments, sites, years, the change trends in LAD and Nac are similar. LAD and Nac increased with increasing N application, the response of LAD and Nac to N treatment varied with N split applications and topdressing time in different experiments. Topdressing time also showed significant effects on LAD and Nac. The LAD ranged from 0.43 to 1.82, 0.46 to 1.824, 0.075 to 2.361, and 0.046 to 1.317 while Nac ranged from 0.67 to 3.7%, 0.83 to 3.87%, 0.95 to 3.35%, and 1.02 to 4.13% in Experiment 1–4, respectively. The LAD of cultivar Aikang58 in Xinxiang (Experiment 3) was higher than that of experiments in Yizheng (Experiments 1, 2 and 4). However, the variation of Nac in different experiments was minor.

**Table 2:** Nitrogen concentration at different growth stages and varied N rates.

**Experiment**	**Treatment**	**Growth stage**									
		**Spring re-growth**		**Jointing**		**Booting**		**Heading**		**Filling**	
		**Nac**	**LAD**	**Nac**	**LAD**	**Nac**	**LAD**	**Nac**	**LAD**	**Nac**	**LAD**
Exp. 1 (Yizheng, 2010–2011)	N0	2.89c	0.043d	2.23d	0.110d	1.14d	0.344d	0.88d	0.525d	0.67c	0.695d
	N1R1	3.21b	0.056c	2.57bc	0.152c	1.57c	0.509c	1.65a	0.886c	1.09ab	1.339c
	N1R2	3.24b	0.061b	2.82b	0.166c	1.82b	0.583bc	1.46b	0.992b	1.06b	1.497b
	N1R3	3.34b	0.058bc	2.71b	0.169bc	1.74bc	0.620b	1.27c	1.032b	1.12a	1.521b
	N1R4	3.33b	0.059b	3.06a	0.190b	1.93b	0.633b	1.61a	1.048b	1.05b	1.542b
	N1R5	3.58a	0.061b	3.09a	0.202a	2.21a	0.685b	1.44b	1.115b	1.08ab	1.619b
	N2R1	3.64a	0.059b	2.60bc	0.168bc	1.88b	0.596bc	1.68a	1.020b	1.16a	1.538b
	N2R2	3.60a	0.058bc	2.54c	0.189b	2.21a	0.642b	1.53ab	1.077b	1.09a	1.583b
	N2R3	3.65a	0.062ab	3.17a	0.205a	2.18a	0.696a	1.61a	1.179a	1.05b	1.706ab
	N2R4	3.61a	0.063a	2.96ab	0.217a	2.01ab	0.767a	1.57a	1.280a	1.08ab	1.844a
	N2R5	3.70a	0.068a	3.14a	0.223a	2.08ab	0.775a	1.61a	1.275a	1.10a	1.820a
F prob. LSD		^*^	^*^	^*^	^*^	^*^	^*^	^*^	^*^	^*^	^*^
		0.132	0.005	0.274	0.021	0.221	0.078	0.151	0.091	0.062	0.172
Exp. 2 (Yizheng, 2010–2011)	N0	3.07c	0.046c	2.05c	0.111c	1.05d	0.314c	0.94c	0.485c	0.83c	0.672c
	N1R1T1	3.71ab	0.056b	2.58b	0.176b	1.82b	0.607b	1.47b	1.003b	1.16b	1.472b
	N1R1T2	3.48b	0.045c	2.50b	0.158b	1.79b	0.576b	1.46b	0.944b	1.10b	1.348
	N1R1T3	3.47b	0.067a	2.45b	0.196a	1.49c	0.594b	1.42b	0.922b	1.06b	1.284b
	N1R2T1	3.93a	0.050bc	3.06a	0.172b	2.12a	0.650ab	1.73a	1.059b	1.34a	1.537b
	N1R2T2	3.63b	0.062a	2.88a	0.198a	1.84b	0.653ab	1.64a	1.058b	1.29a	1.561b
	N1R2T3	3.84a	0.057b	3.04a	0.192a	1.54c	0.625b	1.51b	0.979b	1.07b	1.425b
	N2R1T1	3.81a	0.068a	2.83a	0.196a	2.39a	0.713a	1.55b	1.224a	1.37a	1.813a
	N2R1T2	3.56b	0.062a	2.89a	0.192a	1.79b	0.673ab	1.66a	1.104ab	1.36a	1.573b
	N2R1T3	4.03a	0.057b	2.79a	0.195a	1.59c	0.639b	1.51b	1.009b	1.27a	1.464b
	N2R2T1	3.80a	0.067a	2.89a	0.216a	2.13a	0.768a	1.62a	1.297a	1.38a	1.966a
	N2R2T2	3.89a	0.061a	2.84a	0.205a	1.82b	0.716a	1.65a	1.212a	1.39a	1.854a
	N2R2T3	3.87a	0.063a	2.96a	0.224a	1.73b	0.732a	1.66a	1.218a	1.27a	1.824a
F prob. LSD		^*^	^*^	^*^	^*^	^*^	^*^	^*^	^*^	^*^	^*^
		0.241	0.007	0.224	0.035	0.236	0.087	0.071	0.193	0.172	0.284
Exp. 3 (Xinxiang, 2011–2012)	N0	2.86d	0.075c	2.77c	0.143c	1.34d	0.612c	1.38d	0.852c	0.95c	1.303d
	N1R1	2.96c	0.092b	2.50*c*d	0.175b	1.71c	0.780b	1.47d	1.093b	1.09b	1.657c
	N1R2	2.96c	0.097b	2.37d	0.178b	1.47d	0.801b	1.49*c*d	1.105b	0.97c	1.682bc
	N1R3	3.01bc	0.102b	2.85b	0.188ab	1.63c	0.857b	1.82b	1.201b	1.04b	1.872b
	N2R1	3.03bc	0.084c	2.85b	0.166b	1.97b	0.796b	1.61c	1.175b	1.18a	1.933b
	N2R2	3.08b	0.100b	2.55*c*d	0.191ab	1.90b	0.879b	1.83b	1.251b	1.12a	2.010ab
	N2R3	3.24a	0.105b	3.12a	0.202a	1.77c	0.909ab	1.75b	1.298ab	1.00bc	2.097ab
	N3R1	2.92*c*d	0.102b	2.85b	0.189ab	2.22a	0.929ab	2.06a	1.348a	1.23a	2.240a
	N3R2	3.30a	0.105b	2.82b	0.204a	1.98b	0.969a	2.04a	1.398a	1.08b	2.317a
	N3R3	3.29a	0.113a	2.91ab	0.201a	1.98b	1.016a	1.93b	1.452a	1.20a	2.292a
	N4R1	3.24a	0.115a	2.65c	0.222a	2.06ab	1.005a	2.13a	1.421a	1.16a	2.273a
	N4R2	3.31a	0.112a	3.06a	0.217a	2.26a	1.063a	1.98ab	1.523a	1.12ab	2.415a
	N4R3	3.35a	0.115a	3.02a	0.217a	2.32a	1.051a	2.16a	1.509a	1.16a	2.361a
F prob. LSD		^*^	^*^	^*^	^*^	^*^	^*^	^*^	^*^	^*^	^*^
		0.087	0.008	0.143	0.029	0.212	0.096	0.117	0.272	0.081	0.265
Exp. 4 (Yizheng, 2010–2011)	N0	2.71d	0.046d	1.74c	0.066d	1.05d	0.320d	1.02d	0.422d	–	–
	N1	3.22c	0.119c	2.03b	0.149c	1.46bc	0.473c	1.10d	0.614c	–	–
	N2	3.80ab	0.170b	2.10b	0.216b	1.60b	0.739b	1.37c	0.975b	–	–
	N3	3.87ab	0.210a	2.18b	0.277a	1.83b	0.966a	1.75b	1.276a	–	–
	N4	4.13a	0.196a	2.60a	0.262a	2.47a	0.962a	1.91a	1.284a	–	–
	N5	3.65b	0.228a	2.95a	0.295a	2.51a	0.991a	2.01a	1.317a	–	–
F prob. LSD		^*^	^*^	^*^	^*^	^*^	^*^	^*^	^*^	–	–
		0.313	0.034	0.394	0.049	0.397	0.221	0.182	0.189	–	–

*LAD, leaf area duration; Nac, shoot nitrogen concentration; LSD, least significant difference*.

* *F statistic significant at the 0.05 probability level. Data within a column followed by a different letter are significantly different (P < 0.05)*.

### Determination of the LAD-based critical nitrogen dilution curve

**Figure 1:**
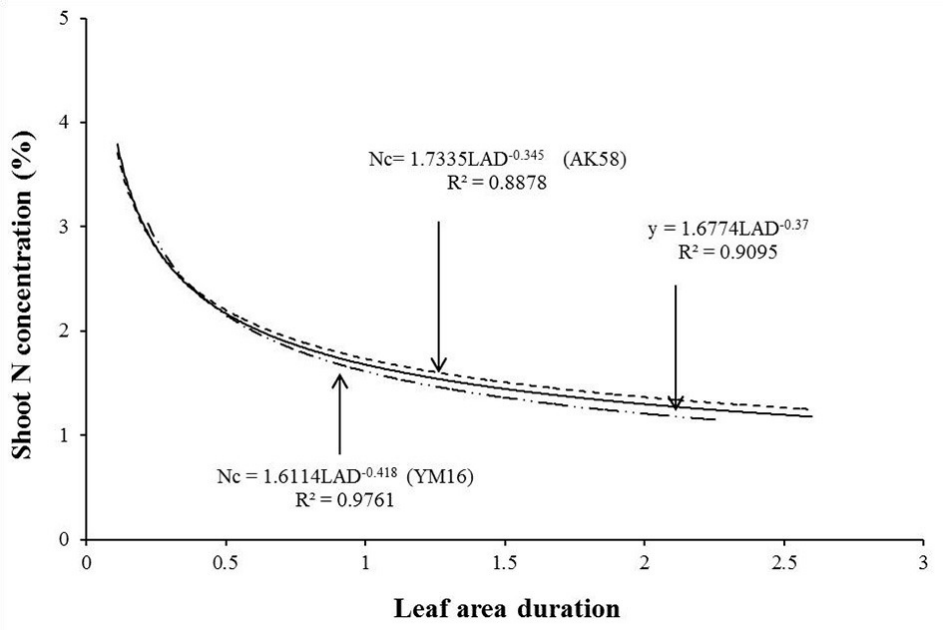
Determination of the LAD-based shoot critical concentration dilution curve for two wheat cultivars using the data of Experiment 1 and 3.

Data from experiments 1 and 3 were used to develop the Nc dilution curve following the method of Justes et al. (1994). Growth rate and cultivar did not significantly affect Nc at both study sites, and the curves for two cultivars showed no significant differences. The unified dilution curve was determined as showed in Figure 1. The constant value of Nc (3.57) is calculated by the equation when LAD = 0.13 when LAD ≤ 0.13. When LAD > 0.13, Nc was calculated as follows.

$$\begin{array}{l} {\text{N}}_{c}=1.6774\;{\text{LAD}}^{-0.37}\;\text{LAD}>0.13\;({\text{R}}^{2}=0.9095)\\ {\text{N}}_{c}=3.57\text{ LAD}\leq 0.13 \end{array}$$

### Validation and application of the established critical nitrogen dilution curve

**Figure 2:**
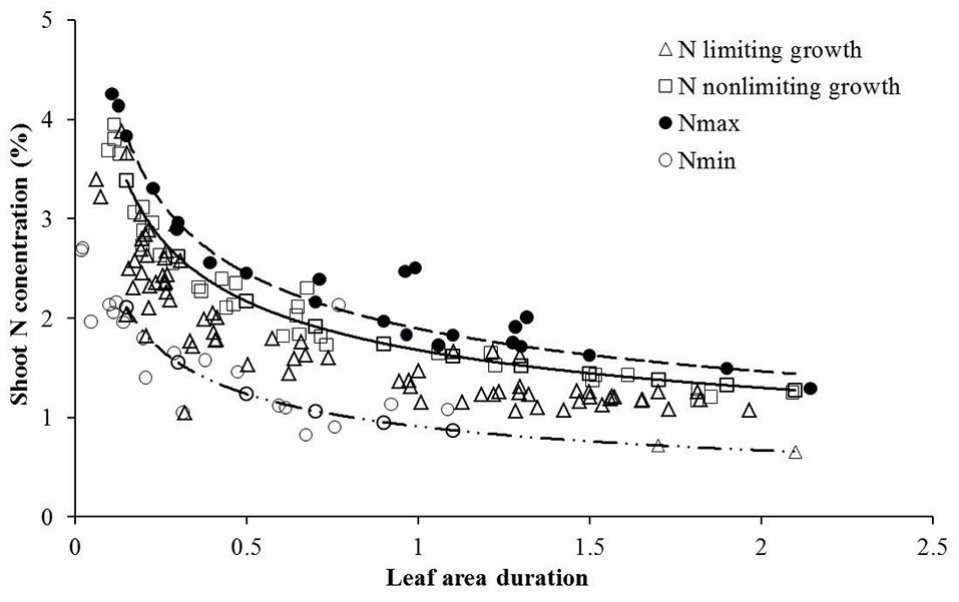
Validation of the Nc dilution curves for winter wheat. Data points (Δ) represent N limiting condition, (□) represent non-N-limiting condition. The solid line indicates the Nc curves (Nc = 1.6774 LAID–0.37, while the dashed lines represent the minimum and maximum N curves respectively, (Nmin = 0.9089 LAD^−0.444^, Nmax = 1.8934 LAD^−0.372^). The (•) points represent N concentration from highest N rate and the (°) points are from zero N rate obtained from Experiments 2 and 4.

The LAD-based Nc dilution curve determined in this study was validated with data acquired from independent experiments (Experiment 2 and 4) using a standard method (Justes et al., 1994; Ziadi et al., 2008, 2010; Zhao B. et al., 2014). The data points under various N treatments from Experiment 2 and 4 were characterized by sub-optimal N or supra-optimal N growth conditions based on significant (*P* ≤ 0.001) differences in shoot biomass for each sampling date, site, and year. Treatments were considered N-limiting when shoot biomass increased significantly with increasing N supply, the N treatment is considered as sub-optimal N supply, while under supra-optimal N supply had no significant increase in biomass with increasing N supply (LSD < 0.05). Data points attained from sub-optimal treatments were located almost below the Nc curve while those of supra-optimal N supply was located close to or above the Nc curve (Figure 2). The newly established LAD-based Nc dilution curve well distinguished the sub-optimal and supra-optimal N supply conditions. Therefore, it was concluded that the curve could be used for in-season assessment of wheat N nutrition status. Data points were selected only from non-N-limiting treatments for the determination of upper limit curve (Nmax) while the data points from N-limiting treatments for which have no N application were used to determine lower limit curve (Nmin) (Figure 2).

### Determination of plant N deficiency

**Figure 3:**
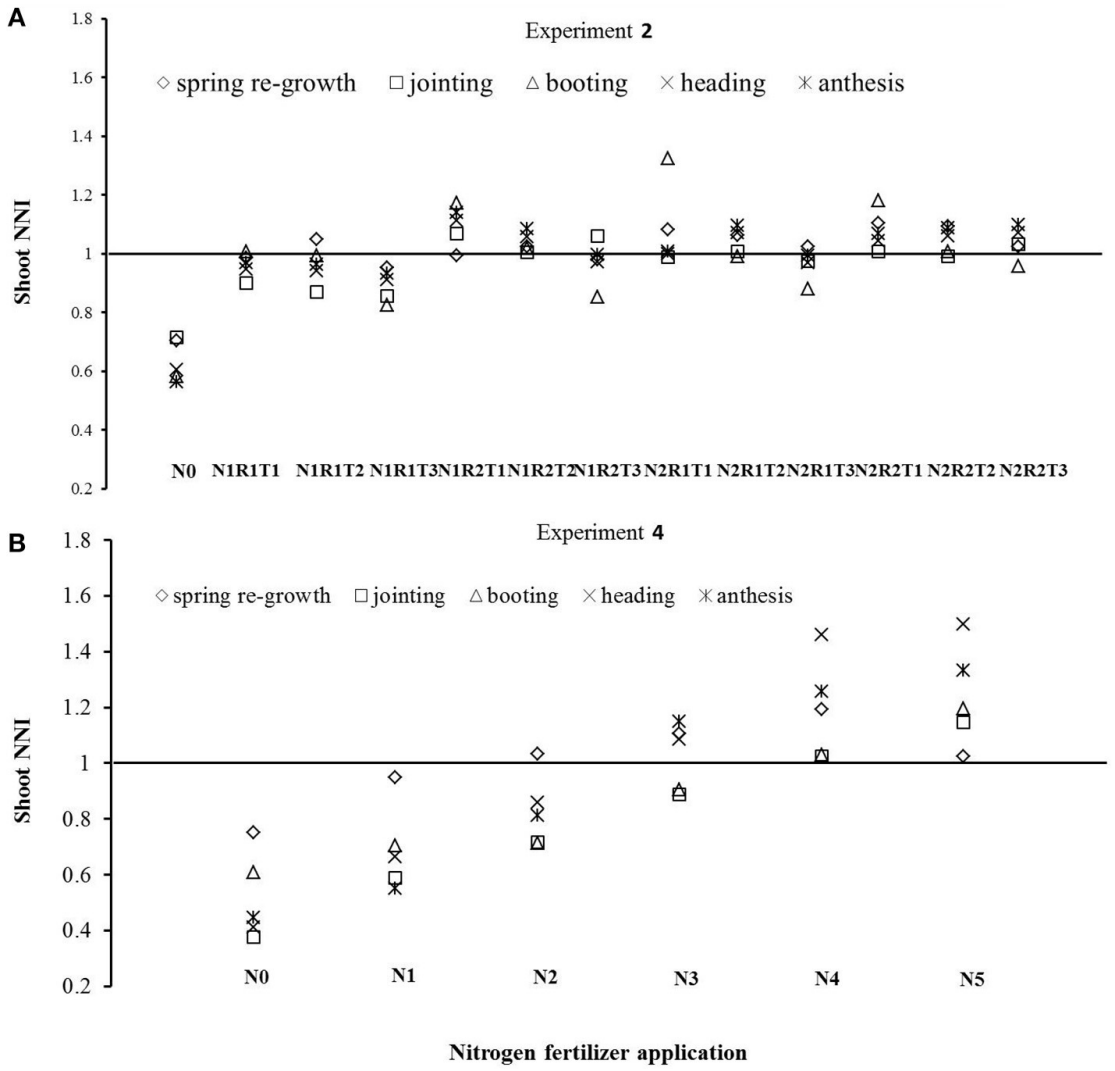
Diagnosis of N status using the LAD-based Nc dilution curves developed in Experiment 2 **(A)** and 4 **(B)**.

Two N application levels in Experiment 2 were relatively high (225 and 300 kg ha^−1^). However, some N split applications still showed N deficiencies at a few growth stages (Figure 3A). Treatments N1R1T1, N1R1T2, N1R1T3, N1R2T3, and N2R1T3 were N deficient at few developmental stages when the base fertilizer level was relatively lower (R1 treatments), or the topdressing was applied later (T3 treatments). In Experiment 4, shoot N concentration under N4, and N5 treatments was significantly higher than shoot Nc. On the other hand, shoot N concentration under N3 was close to the Nc. However, N0, N1, and N2 showed significant N deficiencies due to low levels of N application (Figure 3B). The Nc fell approximately between N3 and N4 levels.

### Relationships between NNI_inte_ and crop growth variables

**Figure 4:**
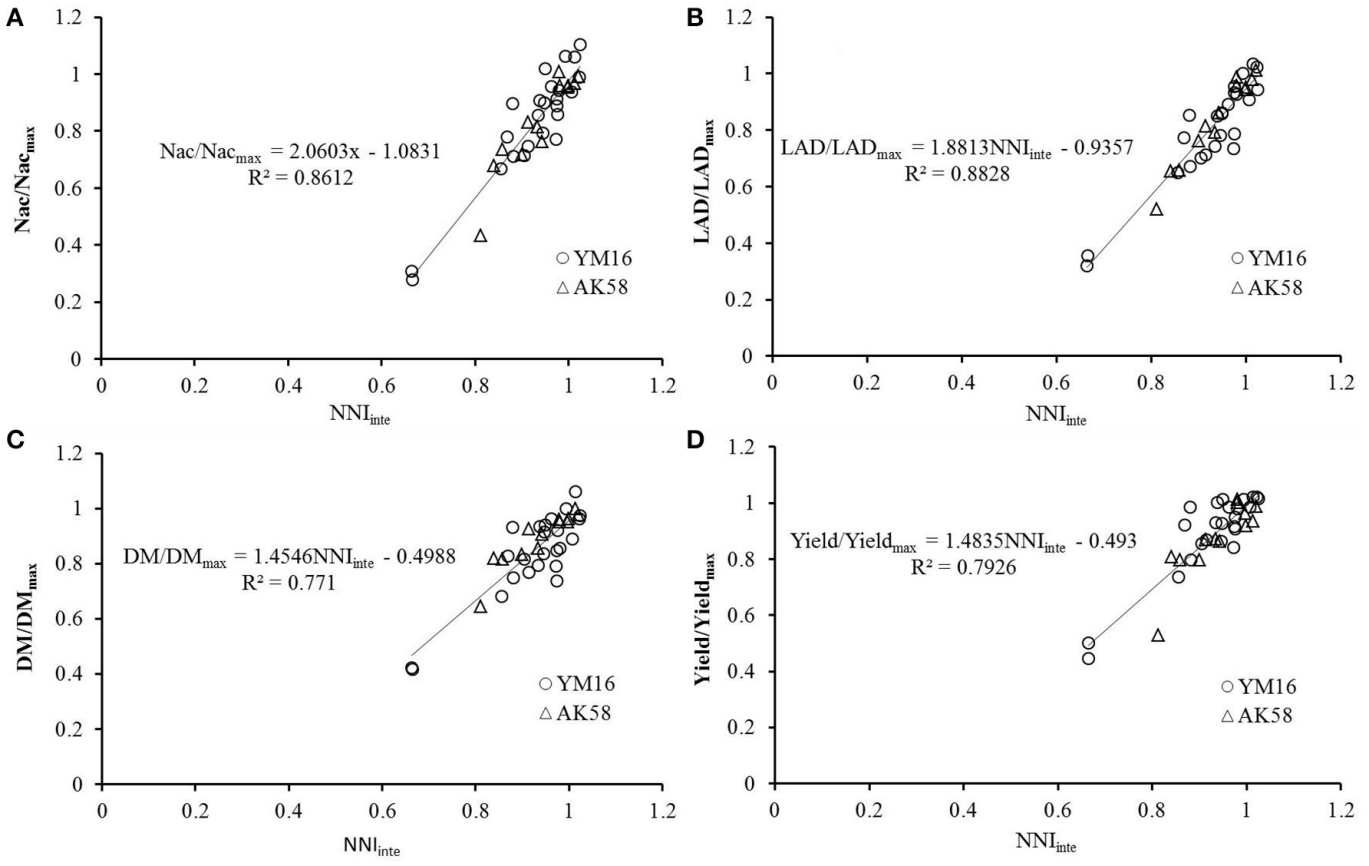
Relationships between relative growth variables and integrated nitrogen nutrition index (NNIinte). **(A)** Nac/Nac_max_; **(B)** LAD/LAD_max_; **(C)** DM/DM_max_; **(D)** yield/yield_max_.

The effects of N deficiencies on growth variables, i.e., dry matter(DM), shoot nitrogen concentration (Nac), LAD and yield was expressed in relative values (DM/DM_max_, Nac/Nac_max_, LAD/LAD_max_, and yield/yield_max_) in order to account for variation in pedoclimatic conditions across the different experiments. The Experiment 1 and 3 were used to develop the linear relationship. DM/DM_max_ represents the ratio between DM accumulated by a crop receiving a limiting N supply and the maximum shoot biomass value measured at the same moment in time, assuming that the maximum biomass (DM_max_) was accumulated under optimum N supply. DM_max_ was calculated as the mean of DM for the group of treatments giving the highest DM value, and not significantly different from each other, at a 5% significance level. The same methodology was applied to calculate DM/DM_max_, Nac/Nac_max_, LAD/LAD_max_, and yield/yield_max_. All of the relative growth variables strongly correlated with NNI_inte_ (Figure 4). The data of experiment 2 was used to validate the relationships between the NNIinte and the relative values (DM/DM_max_, Nac/Nac_max_, LAD/LAD_max_, and yield/yield_max_). The results showed that RRMSE, agreement index, and R^2^ ranged from 6.61% to 11.99%, 0.9515 to 0.9829, and 0.7862 to 0.8968 (Table 3), which indicated that the established relationships are robust. The variation in NNI_inte_ explained more than 70% of variability associated with any of these four variables. When NNI_inte_ equaled 0.80, Nac, LAD, DM, and yield decreased by 42.17, 39.79, 30.44, and 29.95%, respectively.

**Table 3:** Validation of the relationships between the relative growth variables and NNI_inte_.

**Equation**	**RRMSE (%)**	**Agreement index**	**Pr > F**	***R*^2^**
Nac/Nac_max_ = 2.0603 NNI_inte_ −1.0831	9.83	0.9753	<0.0001	0.7862
LAD/LAD_max_ = 1.8813 NNI_inte_ −0.9357	11.94	0.9672	<0.0001	0.8193
DM/DM_max_ = 1.4546 NNI_inte_ −0.4988	11.99	0.9515	<0.0001	0.7862
Yield/Yield_max_ = 1.4835 NNI_inte_ −0.493	6.61	0.9829	<0.0001	0.8968

*Nac, shoot nitrogen Concentration; LAD, leaf area duration; DM, shoot dry matter*.

## Discussion

Crop photosynthetic production capacity is limited by photosynthetic area (which can be quantified through LAI measurements), the duration of the photosynthetic area, and photosynthetic rate. Plant biomass production can be estimated by measuring LAD, which integrated the size and duration of the photosynthetic area (Gastal and Bélanger, 1993; Hammer and Wright, 1994; Zhao et al., 2005). In this study, LAD increased with the increase of N levels while shoot nitrogen concentration decreased with the N levels (Table 2). Similar results for shoot biomass responses to development time and N levels have been reported in previous studies on wheat (Justes et al., 1994; Zhao et al., 2012), rice (He et al., 2017), and maize (Plénet and Lemaire, 1999).

**Figure 5:**
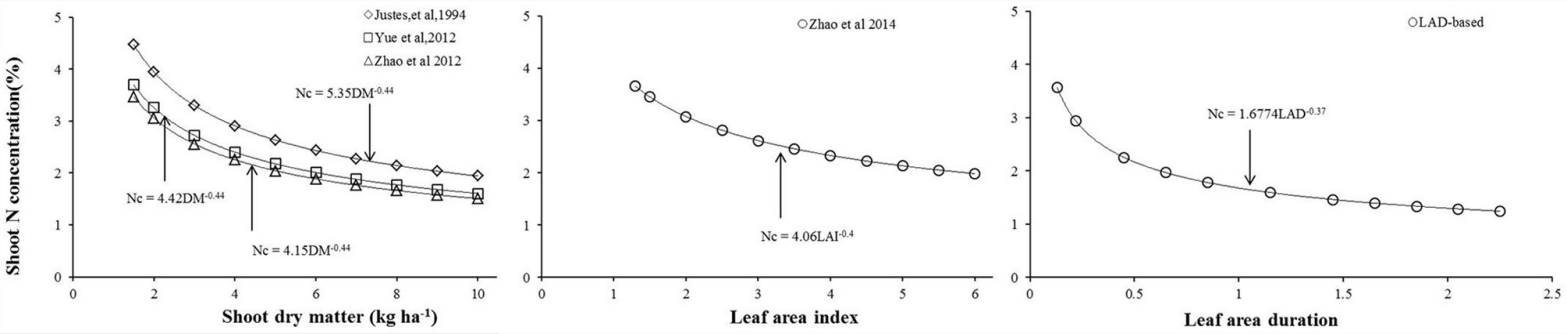
Comparisons of critical dilution curves based on different methods.

Most wheat Nc dilution curves have been developed based on dry matter accumulation (Justes et al., 1994; Yue et al., 2012; Zhao et al., 2012), while Zhao B. et al. (2014) developed a Nc dilution curve based on LAI in winter wheat (Figure 5). This study developed a new LAD-based Nc dilution curve by using the data collected through experiments within the Yangtze river and Yellow river basins of China. Comparing with dry matter-based Nc dilution curves (Justes et al., 1994; Yue et al., 2012; Zhao et al., 2012), LAD explains the process of dry matter accumulation, which can be effected by the size and duration of photosynthetic area. Therefore, the rationales for these two Nc dilution curves types are the same. To account for variations in thermal conditions among the different regions and seasons, thermal time was taken into account for calculating LAD in our study. Additionally, LAI is more easily obtained than shoot dry matter due to most of the instruments designed for measuring LAI. Compared to the LAI-based Nc curve, LAD-based curve can well describe the N dilution during the entire crop growth period, while LAI-based curve only quantifies the Nc dynamics till heading stage (when LAI reaches to its maximum value) (Ata-Ul-Karim et al., 2014b; Zhao B. et al., 2014). Due to the decline of LAI during post anthesis growth period, it does not take the shape of an “S” type curve, hence cannot be used as an appropriate plant index for the elucidation of plant N dilution. Therefore, it is not appropriate to integrate the Nc curves into the crop growth models, especially those which use the Nc concentration of entire crop growth period for simulation of plant N dynamics, such as CERES-wheat (Ritchie and Otter, 1985) and APSIM-wheat (Zhao Z. et al., 2014). Thus, LAD-based Nc curve combines the merits of LAI-based and dry matter-based Nc curve. The initial N concentration in the Nc dilution curve developed in present study was close to that determined by Zhao et al. (2012); yet, our results of N concentration (for a low to middle grain protein cultivar) showed significant differences in the N concentration of high grain protein cultivars used by Yue et al. (2014) and Justes et al. (1994). High grain protein cultivars tend to have better rooting capabilities, allowing uptake and assimilation of more N. Thus, more cultivars should be tested to validate this methodology.

The NNI at various wheat growth stages was calculated using the LAD-based Nc dilution curve. As N treatment levels were applied with different splits and top-dressing stages, there were multiple NNI for each N treatment level (Figure 3). NNI values, which can be used for real-time diagnosis of plant N nutrition status, change with time. Due to different N application modes and soil N mineralization, soil N supply capacity changes constantly, the value of instantaneous NNI was highly variable (Figure 3) and could not be applied to detect N deficiency throughout the entire growth period (Lemaire et al., 2008). NNI_inte_ incorporates the knowledge of time course of the NNI during this period (Lemaire and Gastal, 1997; Colnenne et al., 2002). The linear correlations between NNI_inte_ and DM, LAD, Nac, and yield, were very well (Figure 4). The slopes for the relationships between NNI_inte_ and DM and yield were lower than those for the relationships between NNI_inte_ and Nac and LAI, which suggests that the effects of NNI_inte_ on Nac and LAD were greater and more sensitive to N deficiency than the effects on DM and yield. These relationships between NNI_inte_ and DM, LAD, Nac, and yield were similar to those reported in a previous study (Colnenne et al., 2002; Lemaire et al., 2008).

## Conclusions

This study has developed a new N_*c*_ dilution curve based on LAD in winter wheat, which combines the merits of shoot dry matter- and LAI-based Nc curve. The results showed that the allometric relationships between N uptake and LAD were robust across the different conditions and was described as Nc = 1.6774LAD^−0.37^ (LAD > 0.13), and Nc = 3.57, when LAD ≤ 0.13. NNI derived from the curve established here, effectively identified the limiting and non-limiting N nutrition at different growth stages. The NNI_inte_, which was based on Nc, was applied to regression functions to estimate Nc, shoot dry matter, actual shoot N content, LAD, and yield measured in independent experiments at different growth periods. These relationships showed strong linear correlations between NNI_inte_ and the growth variables. Our results indicated that the developed Nc curve provide a new procedure and alternate method to assess crop N status for N diagnose and management.

## Author contributions

XW and LT wrote the manuscript; XW analyzed the experiments data; SA, YZ and WC provided advice and edited the manuscript; LT, WC, and YZ planned experiments and XW, TY, and LL performed experiments. All authors read and approved the final manuscript.

### Conflict of interest statement

The authors declare that the research was conducted in the absence of any commercial or financial relationships that could be construed as a potential conflict of interest.
